# Digital implantology—a review of virtual planning software for guided implant surgery. Part II: Prosthetic set-up and virtual implant planning

**DOI:** 10.1186/s12903-022-02057-w

**Published:** 2022-01-30

**Authors:** Tabea Flügge, Jaap Kramer, Katja Nelson, Susanne Nahles, Florian Kernen

**Affiliations:** 1 Corporate Member of Freie Universität Berlin and Humboldt Universität Zu Berlin, Charité – Universitätsmedizin Berlin, Berlin, Germany; 2 Department of Oral and Maxillofacial Surgery, Translational Implantology, Medical Center – University of Freiburg, Faculty of Medicine, University of Freiburg, Freiburg, Germany

**Keywords:** Guided implant surgery, Computer-assisted surgery, Computer-aided design, Virtual implant planning

## Abstract

**Background:**

Patient- and technology-related parameters influence the successful implementation of virtual implant planning and guided implant surgery. Besides data processing and computer aided design of drill guides as described in Part I, the possibilities and limitations for prosthetic set-up and virtual implant planning are essential (Part II).

**Methods:**

The following software systems were examined using two different clinical situations for implant therapy: coDiagnostiX™, DentalWings, Canada (CDX); Simplant Pro™, Dentsply, Sweden (SIM); Smop™, Swissmeda, Switzerland (SMP); NobelClinician™, Nobel Biocare, Switzerland (NC); Implant Studio, 3Shape, Denmark (IST). Assessment criteria geared towards interfaces and integrated tools for prosthetic set-up and virtual implant planning.

**Results:**

A software interface for an individual virtual prosthetic set-up was provided by two systems (CDX, IST), whereas the set-up of standardized teeth was provided by four systems (CDX, SIM, SMP, IST). Alternatively, a conventional set-up could be scanned and imported. One system could solely work with the digitization of a conventional set-up for virtual implant planning (NC). Stock abutments could be displayed for implant planning, but none of the tested software systems provided tools for the design of an individual abutment. All systems displayed three-dimensional reconstructions or two-dimensional cross-sections with varying orientation for virtual implant placement. The inferior alveolar nerve could be marked to respect a minimum distance between the nerve and the planned implant. Three implant planning systems provided a library to display more than 50 implant systems (CDX, SIM, IST), one system provided 33 implant systems (SMP) and one implant system provided 4 implant systems (NC).

**Conclusion:**

Depending on the used software system, there are limited options for a virtual set-up, virtual articulators and the display of a virtual prosthetic set-up. The implant systems used by the clinician is important for the decision which software system to choose, as there is a discrepancy between available implant systems and the number of supported systems in each software.

**Supplementary Information:**

The online version contains supplementary material available at 10.1186/s12903-022-02057-w.

## Background

**Fig. 1:**
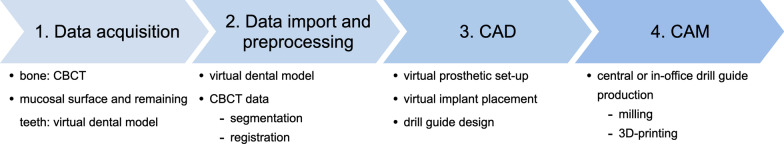
Digital Workflow for preoperative planning for guided implant surgery

The digital workflow of preoperative implant planning for guided implant surgery includes digital data acquisition and processing, computer-aided design (CAD) and computer-aided manufacturing (CAM) (Fig. 1).

With dedicated software systems, the prosthetic set-up is virtually designed and implants are positioned regarding the set-up and individual anatomy to achieve an ideal implant position [1]. The planned implant position is transferred into surgery with a virtually designed drill guide, which is produced in a production center, specialized dental laboratory or in-office.

Data acquisition, import and visualization as well as drill guide design and manufacturing are covered in Part I, the prosthetic set-up and virtual implant planning are examined in the present Part II of the narrative review [2].

### Prosthetic set-up

Ideal implant placement is not only defined by the osseous anatomy, but the planned implant-supported prosthesis with its final position and characteristics including crown morphology, emergence profile, occlusal and proximal contacts [3]. A correct implant position entails a favorable esthetic outcome and facilitates optimal occlusion and implant loading for biomechanical and functional stability [4]. Prosthetic factors regarding implant planning are summarized in Table 1.

**Table 1:** Prosthetic factors to be considered for precise virtual implant planning

Prosthetic factors	Final position of implant-supported prosthesis
	Crown morphology
	Occlusal contacts
	Proximal contacts
	Abutment design (pink-white esthetics, emergence profile)

**Fig. 2:**
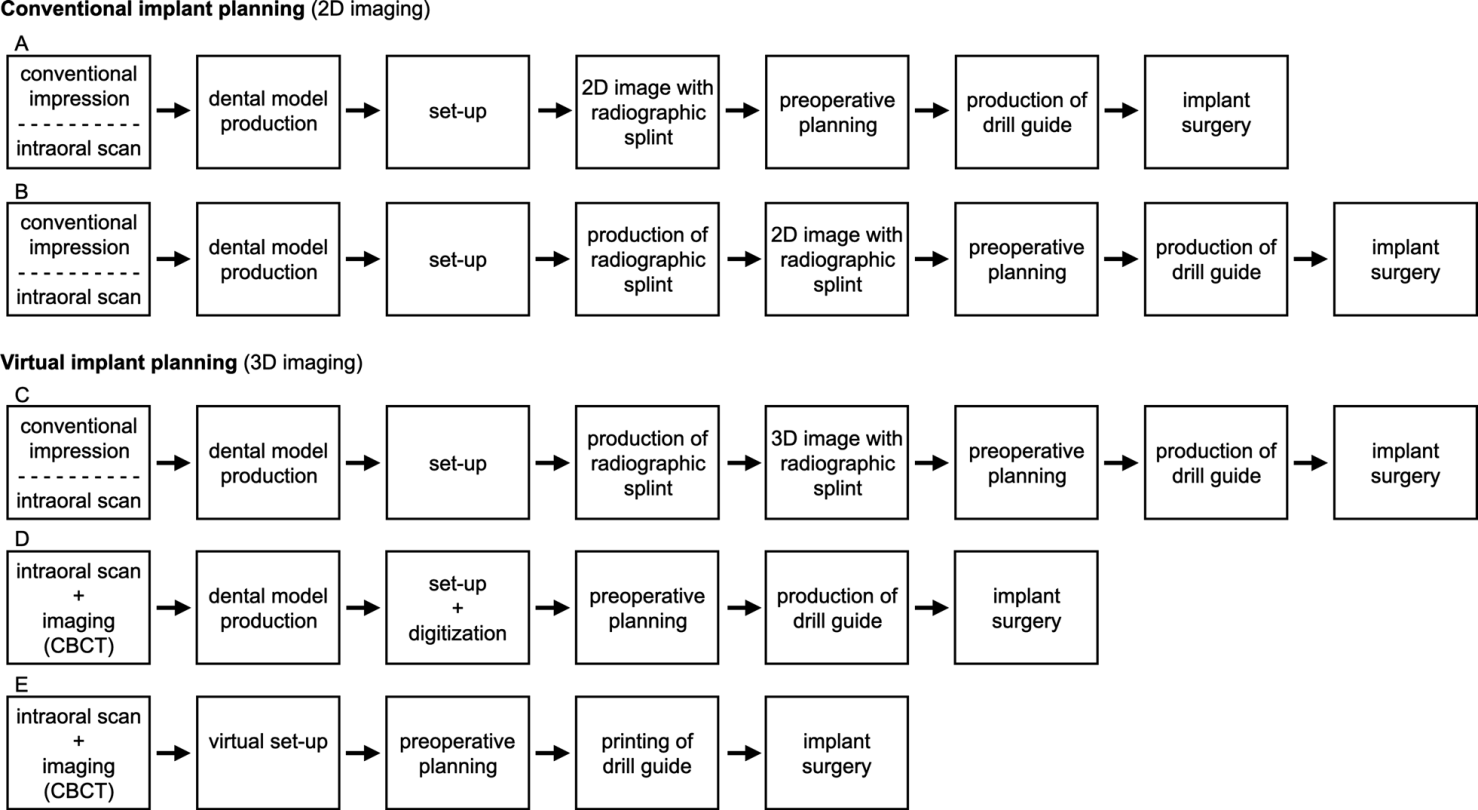
Available workflows for preoperative implant planning. **A** Conventional implant planning with a conventional prosthetic set-up on stone casts of patients without 3D image data acquisition. **B** and **C** Virtual implant planning with a prosthetic set-up fabricated on individual stone cast being transferred to a radiographic splint. **D** Virtual implant planning with the prosthetic set-up being optically scanned along with the stone cast and registered with the radiographic data for subsequent planning. The digitization requires no preliminary radiographic splint as prior steps are performed within the software. **E** Virtual implant planning with a virtual prosthetic set-up requiring no conventional set-up and radiographic splint resulting in reduced working steps within the complete digital workflow

Traditionally, the planning of an implant-supported prosthesis is based on a set-up fabricated on individual stone casts of the patient (Fig. 2A). The set-up is transferred to a resin splint equipped with radiopaque teeth or sleeves that mark the tooth position [5, 6]. The radiographic splint may be worn during (cone beam) computed tomography (CT or CBCT) to display the restoration in the radiographic volume. Afterwards, the radiographic splint is modified to be used for guided implant surgery [7–9]. In addition, to the prosthetic set-up, radiographic splints carry reference markers to align virtual dental models with radiographic data resulting in double-scan and single-scan protocols depending on the reference markers used [2]. Alternatively, the prosthetic set-up may be optically scanned along with the stone cast and registered with the radiographic data for subsequent planning.

A virtual prosthetic set-up requires no preliminary conventional set-up and no radiographic splint as all steps are performed within the software [10]. Alternatively, the virtual set-up may be imported through an interface after being completed by a laboratory-based software. Within the planning software, varying tools are provided for a virtual set-up. Among these are a library with standard tooth shapes, shaping tools and virtual articulators to realize a functional set-up. Based on a library with multiple tooth shapes, a biogeneric prosthetic set-up was developed to allow for automated design of a restoration in CAD software [11–13]. A biogeneric prosthetic set-up is based on a mathematical algorithm that includes multiple characteristics of teeth to calculate a prosthetic set-up for a missing tooth/missing teeth. Some laboratory-based planning systems already incorporate an algorithm named biogeneric design mode [14].

Virtual articulators have been implemented in CAD software for the analysis of static and dynamic occlusion replacing mechanical articulators. In accordance with mechanical articulators and depending on the chosen virtual articulator, settings such as Bennett angle, condylar inclination and immediate mandibular lateral translation may be adjusted. Further, masticatory movements, e.g. protrusive or lateral excursions, may be simulated. The occlusion is marked in color for automatic or manual alignment of the virtual set-up.

The available options for preoperative implant planning, ranging from conventional to a complete digital workflow, are summarized in Fig. 2.

### Virtual implant planning

Radiographic data of the patient is recorded to obtain information about bone dimensions in the proposed region of implant placement. Three-dimensional (3D) imaging, e.g. CBCT, provides the crucial information for virtual implant planning [3, 15] (Fig. 2B–D).

With the information of the prosthetic set-up and the individual anatomy, implants are virtually positioned in cross-sectional images and three-dimensional surface models reconstructed from the radiographic volume. The software assists with distance measurements between planned implants (3 mm) and between implant and inferior alveolar nerve canal (2–5 mm) [16]. Anatomical factors considered for implant planning are summarized in Table 2.

**Table 2:** Anatomical factors to be considered for precise virtual implant planning

Anatomical factors	Relationship to important anatomical structures (nerves, vessels, roots, nasal floor, sinus cavities)
	Bone quantity (horizontal and vertical)
	Bone quality (cortical/cancellous)
	Contour/amount of soft tissue

The implant planning software should allow the virtual placement of a variety of implant systems by choosing an implant icon from the toolbar which changes the implant type, length, diameter, height, inclination and rotation as often as required. After final selection of an implant, its position is approved in multiplanar images and the corresponding drill sleeves are selected for guided implant surgery [17].

In this narrative review, commercially available systems for virtual implant planning are examined regarding the prosthetic set-up and the integration of anatomical data.

## Methods

Two patients with different clinical situations were used for the examination of the following commercially available virtual implant planning systems: coDiagnostiX, Version 9.9. (DentalWings, Canada) (CDX); Simplant Pro, Version 17 (Dentsply, Sweden) (SIM); Smop, Version 2.13. (Swissmeda, Switzerland) (SMP); NobelClinician, Version 2.4. (Nobel Biocare, Switzerland) (NC); ImplantStudio Version 1.6.4.4, (3Shape, Denmark) (IST).

**Fig. 3:**
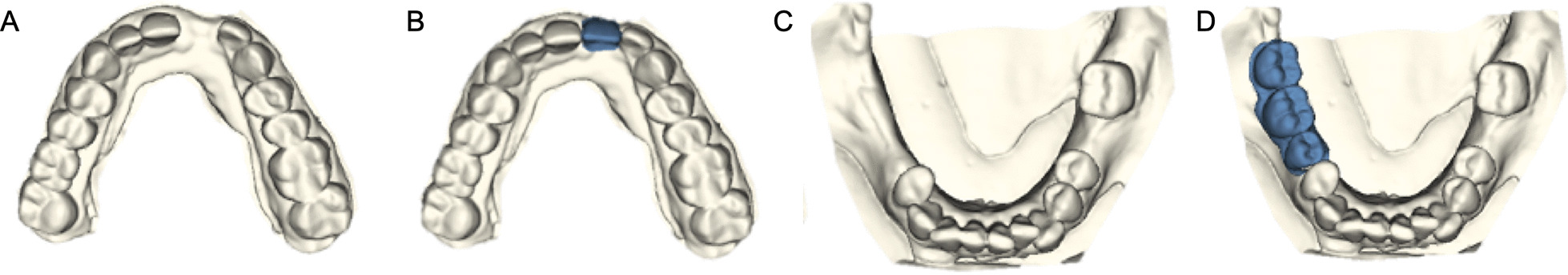
Indications for dental implant treatment. **A** Single-tooth space (FDI region 21), **B** Diagnostic prosthetic set-up for region 21. **C** Right posterior partial edentulism (FDI region 45–47), **D** Diagnostic prosthetic set-up for region 45–47

The patients presented two different indications for dental implant treatment. First patient had a missing tooth in region 21 (FDI); the second patient was partially edentulous in the right mandible with missing teeth 45–47 (FDI) (Fig. 3).

CBCT data and intraoral scans of the first patient (iTero, Cadent, Santa Clara, CA, US) as well as virtual dental model (D250, 3Shape, Copenhagen, Denmark) of the second patient were available. The above-mentioned virtual implant planning systems were evaluated by one examiner with defined assessment criteria.

### Virtual articulator and prosthetic set-up

The import options and integrated tools for a prosthetic set-up and the synergy between design software systems were examined. Implant planning software was reviewed in their availability for an interface to a laboratory software for communication. The availability of a virtual articulator with individual facebow settings (Bennett angle, condylar inclination, immediate mandibular lateral translation) and the simulation of dynamic occlusion were documented (Table 3). Further, the criteria for import options of a prosthetic set-up, such as digitization of a conventional set-up, integration of a conventional set-up using a radiographic splint or virtual set-up following a complete digital workflow, were assessed (see Fig. 2). For the virtual prosthetic set-up, the availability of a library of standard tooth shapes or the possibility to derive a biogeneric prosthetic set-up was tested. With the virtual implant and prosthetic set-up in place, the availability of different types of stock abutments for implant planning were documented. Virtual tools for individualization of the set-up, design and display of an individual abutment were assessed for each software system (Table 3).

**Table 3:** Assessment criteria of the software systems for facebow settings and prosthetic set-up

Virtual articulator	Bennet angle
	Condylar inclination
	Immediate mandibular lateral translation
	Dynamic occlusion
Prosthetic set-up	CBCT scan with/without a radiographic splint
	Interface for individual prosthetic set-up in external CAD-software
	Prosthetic set-up modifiable during planning process
	Tools for digital tooth design
	Biogeneric prosthetic set-up
	Display of stock abutments
	Individual abutment design

### Virtual implant planning

For preoperative implant planning, virtual implants representing the exact dimensions are required. The available implant systems in each software were documented. The default settings for the report of potential violation of minimal distances between multiple implants, implants and adjacent teeth and the inferior alveolar nerve, respectively, were assessed. Measuring tools for distances and angulations between implants and neighboring teeth were evaluated. For detection of bone defects measuring tools for the bone volume in the region of planned implant position were assessed. The assessment criteria of the software systems for preoperative implant planning are outlined in Table 4.

**Table 4:** Criteria for assessment of software systems for virtual implant planning

Virtual implant planning	Implant systems
	Selective display of mandibular canal
	Default settings of minimal distances around implants
	Measurement of bone defect/display of bone augmentation volume

## Results

### Virtual articulator and prosthetic set-up

A virtual prosthetic set-up was offered within all tested implant planning systems; however, not with the full range of possibilities offered by complete Dental-CAD systems that is used for design and production of prosthetic restorations (Table 5).

**Table 5:** Results of the assessment criteria (facebow settings and prosthetic set-up) for the examined commercially available virtual implant planning systems

	CDX	SIM	SMP	NC	IST
*Virtual articulator*					
Bennet angle	X	X	X	X	✓
Condylar inclination	X	X	X	X	✓
Immediate mandibular lateral translation	X	X	X	X	✓
Dynamic occlusion	X	X	X	X	✓
*Prosthetic set-up*					
CBCT scan with radiographic splint	✓	✓	✓	✓	✓
CBCT scan without radiographic splint	✓	✓	✓	✓	✓
Interface for individual prosthetic set-up in external CAD-software	✓	X	X	X	✓
	Cares, Straumann				Dental System, 3Shape
Prosthetic set-up modifiable during planning process	✓	✓	✓	X	✓
Tools for digital tooth design	✓	✓	(✓)	X	✓
Biogeneric prosthetic set-up	X	X	X	X	X
Individual abutment design	X	X	X	X	X
Display of stock abutments	✓	✓	✓	X	✓

**Fig. 4:**
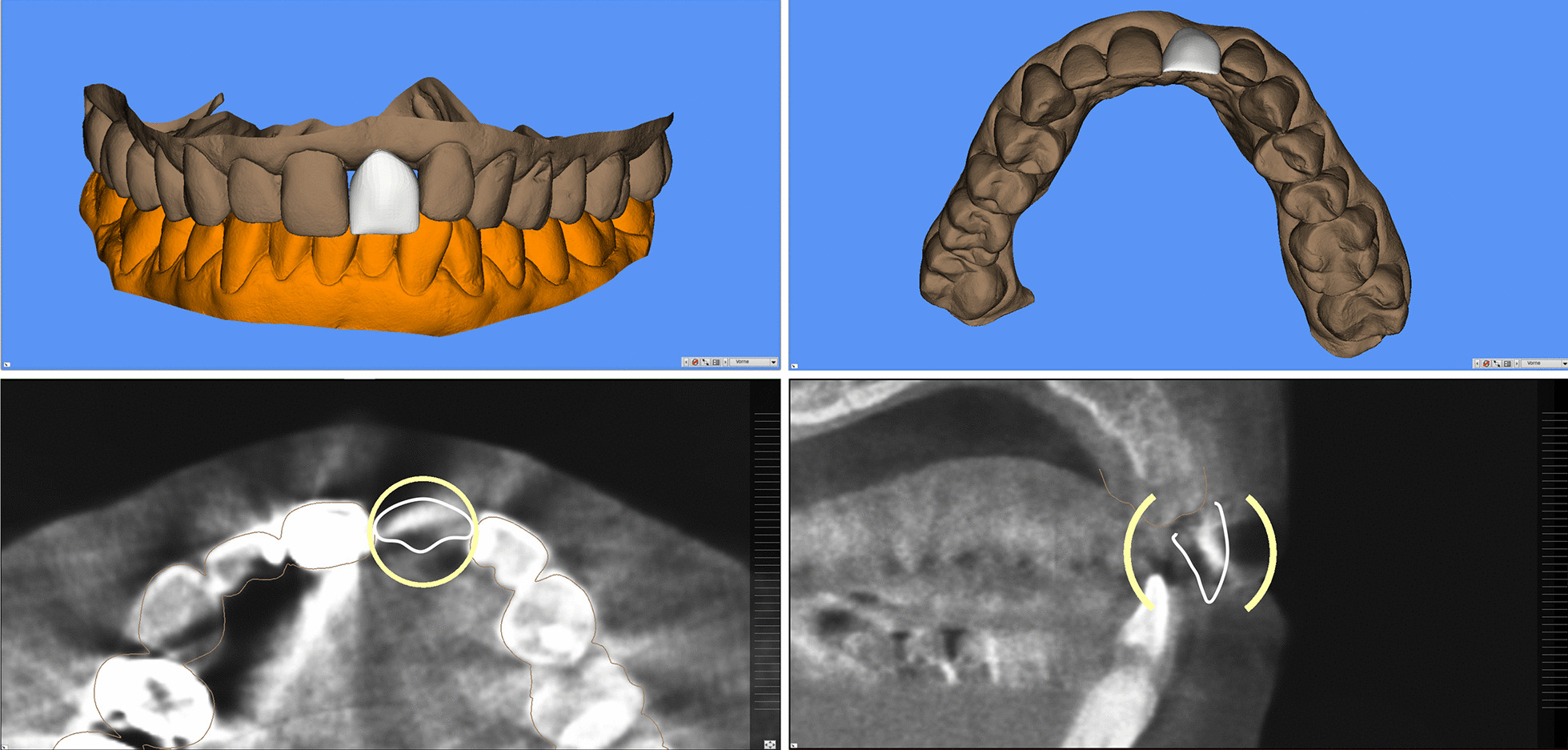
Prosthetic set-up of the restoration within the implant planning software Smop (SMP) using a standard tooth from a library to be fitted in the edentulous space

A library with various tooth shapes for the prosthetic set-up was available in implant software systems (CDX, SIM, IST) so that the size and shape of teeth could be aligned and rotated in all spatial directions. However, in one system (SMP) individualization was limited, only the tooth size but not the tooth shape could be adjusted (Fig. 4).

**Fig. 5:**
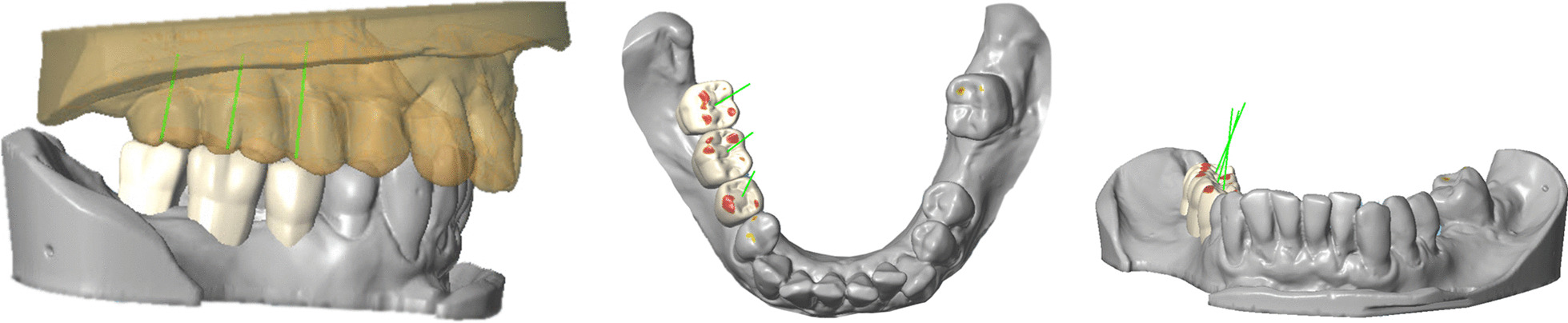
Prosthetic set-up in a full CAD-software (Cares, Dentalwings) using individual tools to create restorations and adapt the occluding surfaces

The selection of different tooth shapes (IST) or the use of a standard tooth shape (CDX, SMP, SIM) was possible. Only one implant system (IST) provided a virtual articulator to simulate the dynamic occlusion and its effect on the virtual set-up. A biogeneric set-up was not available in any of the software systems tested. Two systems (CDX, IST) additionally provided an interface with a full CAD-software (Cares, Straumann AG and Dental System, 3Shape, respectively) (Table 5). These additional software systems allowed to virtually design the set-up and import it into the implant planning software in a proprietary data format (Fig. 5).

**Fig. 6:**
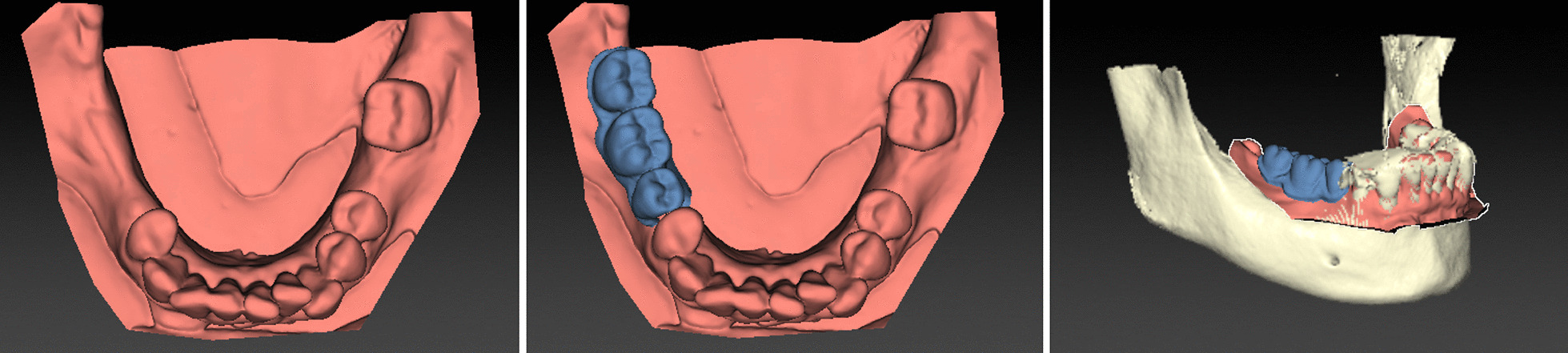
Diagnostic wax-up on a stone cast and subsequent scanning of the diagnostic stone cast and the prosthetic set-up. Display of the prosthetic set-up in the implant planning software NC (Nobel Clinician, Nobel Biocare)

Virtual tools for the design or the adjustment of the prosthetic set-up were not available in one software system (NC), but a conventional prosthetic set-up could be scanned and imported (Fig. 6). A more recent version of NC, today DTX Studio Implant Version 3.5 allows to set-up standardized teeth and additionally offers a software interface for an individual prosthetic set-up.

The import of a conventional prosthetic set-up with subsequent production of a radiographic splint could be realized with all systems tested (CDX, SIM, SMP, NC, IST) (Fig. 2B).

Stock abutments could be displayed for implant planning (CDX, SIM, SMP, IST), however the variety of different stock abutments (tissue/bone level) was limited (SIM, SMP). The user selected an abutment that could be customized to simulate various abutment types and angled abutments respectively (CDX, SIM, SMP). One system used only proprietary implant abutments (NC) for Nobel Biocare implants, although various implant symstems were available in the virtual implant planning. None of the tested software systems provided tools for the design of an individual abutment.

### Virtual implant planning

Virtual positioning of implants was realized in multiplanar, panoramic and three-dimensional reconstructions of CBCT data (CDX, SMP, SIM, NC, IST). Multiplanar reconstructions included axial, transversal and tangential cross-sections, displayed after individual adaptation of a panoramic curve along the alveolar bone (CDX, SIM, SMP, NC, IST).

The software systems varied in the availability of implant systems: Over 50 different manufacturers were listed in CDX, SIM, IST, 33 in SMP, and 4 in NC, respectively. The most recent information about the availability of implant manufacturers are complemented in an additional file for up-to date versions of CDX with 86, SIM with 120, SMP with 52, NC (today DTX Studio Implant) with 7 and IST 100 available implant manufacturers, respectively (see Additional file 1). Fully-guided implant placement or at least guided pilot-drilling was possible for a limited number of implant manufacturers: 26 with CDX, 24 with SIM, 36 with SMP, 1 with NC and 66 with IST, respectively (see Additional file 1).

The intrabony course of the inferior alveolar nerve was recognized semi-automatically by selecting its most anterior and most posterior portions and could be adjusted manually (CDX, SIM, SMP, NC, IST). After segmentation of the inferior alveolar nerve a notification was visible when the implant position was violating a minimal distance of 2 mm around the marked nerve course. A minimum clearance of 2 mm circumferential to the implants was displayed with a box (CDX, SMP, NC, IST) or default settings for the report of potential violation of minimal distances between two implants were available (CDX, SMP, SIM, NC, IST).

**Fig. 7:**
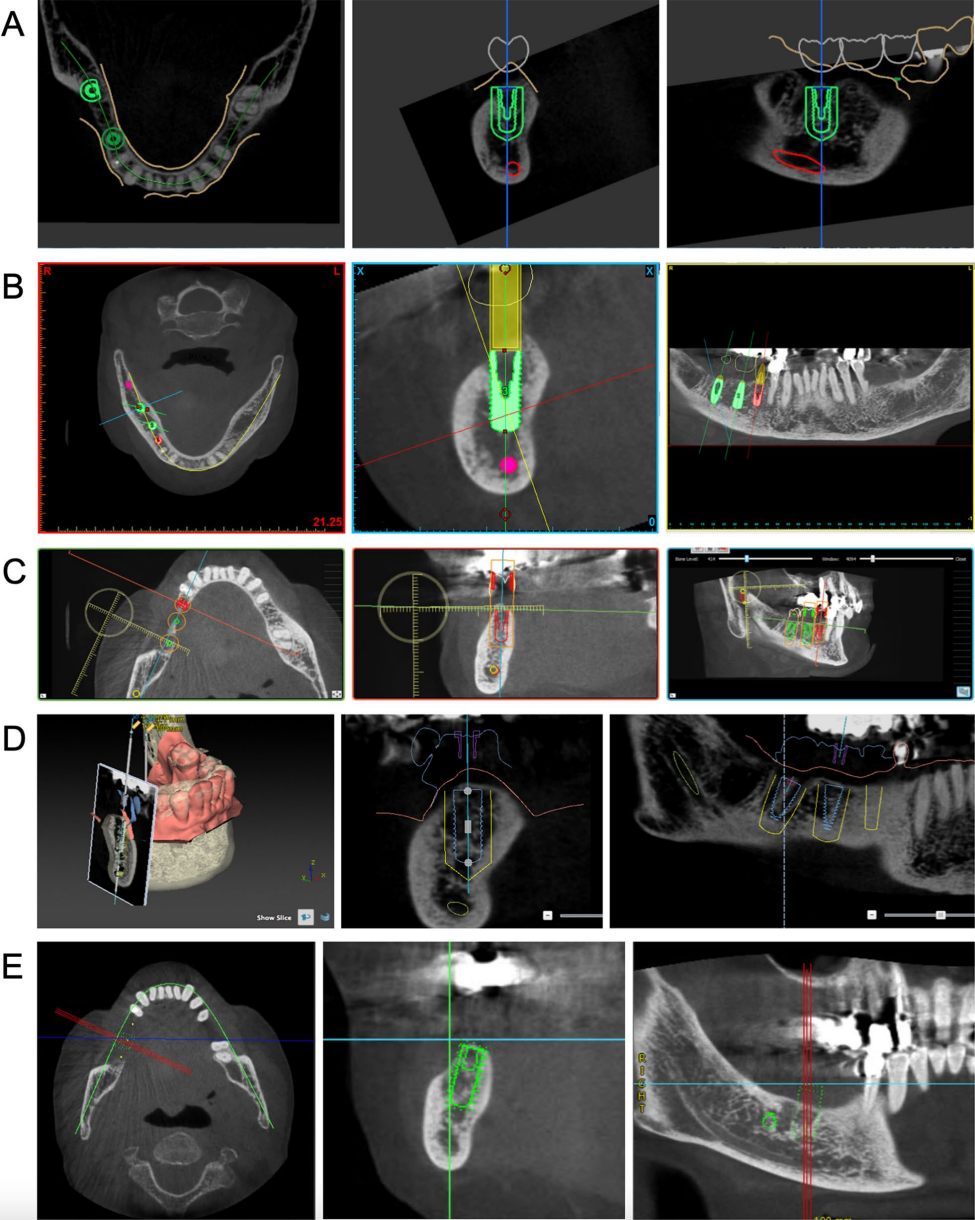
Implant planning with two-dimensional multiplanar reconstructions in Implant Studio (IST) (**A**); Simplant (SIM) (**B**); Smop (SMP) (**C**); NobelClinician (NC) (**D**); coDiagnostiX (CDX) (**E**)

Bony defects at the site of planned implant position could be measured with one system to plan an augmentation and detect the bone augmentation volume (SMP). Options for virtual implant placement are displayed in Table 6 and Fig. 7.

**Table 6:** Display options and planning aids for virtual implant planning with the tested software systems

Virtual implant planning	CDX	SIM	SMP	NC	IST
Implant systems	> 50	> 50	33	4	> 50
Selective display of mandibular canal	✓	✓	✓	✓	✓
Default settings of minimal distances around implants	✓	X	✓	✓	✓
Display of volume for bone augmentation	X	X	✓	X	X

## Discussion

Five commercially available virtual implant planning systems were examined regarding prosthetic set-up and virtual implant planning. Previously, virtual implant planning systems have been assessed regarding their accuracy for guided surgery [3]. The present paper examines and compares the capabilities and limitations of virtual implant planning systems based on defined assessment criteria.

The prosthetic restoration of an implant is as important for its position as the anatomical prerequisites [18, 19]. All examined systems allowed the use of conventional prosthetic set-up and its virtual integration with a radiographic splint [20] or the optical scanning and data import of the set-up [21, 22]. Different workflows for prosthetic set-up and virtual implant planning may be followed (conventional, virtual with/without radiographic splint, complete digital workflow). The complete digital workflow, without a conventional set-up using standard teeth from a virtual library, was only available for four out of five systems [10]. One system therefore only supported a conventional set-up (NC).

In the complete digital workflow only one consultation for radiologic and clinical data acquisition is required to produce an implant drill guide and perform guided implant surgery in the second consultation. Higher efficiency is attained by avoiding conventional intraoral impressions, manufacturing of stone casts, the conventional set-up of teeth as well as the fabrication of a radiographic splint [20]. It can be assumed that transfer errors (e.g. taking a conventional impression, fabricating a stone cast) are avoided using virtual tools for the prosthetic set-up based on imaging data [20].

The goals of preoperative surgical planning in dental implantology are the display of the alveolar bone in the implant region and the identification of anatomical structures important for the implant position [3, 23]. Three-dimensional reconstructions and multiplanar cross-sections, oriented along the alveolar process in the implant region, were available in all systems to review important parameters for the implant position. The individual intrabony course of the inferior alveolar nerve could be marked to detect the distance of the planned implant position to the nerve canal. Warning notifications were issued in case implants were placed below the minimal distance between each other and the inferior alveolar nerve (CDX, SMP, SIM, NC, IST), respectively, and with two systems a frame displayed the leeway around implants (SMP, NC). However, a warning notification for the minimum distance between the implant and adjacent teeth was not available in the implant planning software systems tested, as teeth are not automatically detected by the software. Due to the incapability of recognizing bony surface the software did not allow similar notifications regarding minimal periimplant bone volume. Several software systems include the display of bone densities in the planned implant region. The bone density is calculated on the basis of grey values of the CBCT. Previous studies have shown that grey values in CBCT are not a reliable tool to determine bone density [24]. Therefore, this tool was not included in the presented evaluation of software systems.

The integration of additional surface scans e.g. face scans is not fully applied in the complete digital workflow yet although the facial profile has to be considered for an esthetic result [25]. To date, more recent versions of implant planning systems (CDX, IST, NC) allow the import of patient photographs or face scans, though the registration to the skeletal structures remain challenging.

Virtual design options of the current implant planning software systems differ from full CAD-systems. Within implant planning software fewer tools for the individualization of the prosthetic set-up are available and the virtual prosthetic planning in the CAD-software cannot be exported for the production of a prosthetic framework. So far, only two of the examined systems provided an interface with a full CAD-software and allowed the import of a virtual prosthetic set-up created with the CAD-system (Cares, Straumann AG and Dental System, 3Shape) (CDX, IST).

Dental models are aligned with CBCT data; consequently, the alignment of the dental models follows the bite taken during the CBCT scan. Within the full CAD system virtual models might be aligned in occlusion and individual settings (Bennett angle, condylar inclination, immediate mandibular lateral translation) and thus possibly regarded to simulate the dynamic and static occlusion. The alignment of maxilla and mandible in occlusion was possible in one of the systems tested (IST). Dynamic occlusion could be performed using a virtual articulator to evaluate and adjust the virtual prosthetic set-up.

Moreover, the actual production of an implant abutment and restoration is only available with full-CAD systems. The synergy of full CAD-systems and CAD-formats in virtual implant planning software may facilitate the CAD/CAM workflow. A useful feature would be the application of a virtual prosthetic set-up within a full CAD-software that can be worked with subsequently. 3D virtual articulation systems are currently developed incorporating virtual reality applications for a full analysis of the interoclusal relation, condition of the temporomandibular joint and masticatory movement, but not implemented yet (including force and frequency of occlusal contacts in relation to time) [26, 27]. It remains to be seen if future developments allow a better link between the virtual prosthetic set-up and implant planning for virtual set-up, e.g. biogeneric set-up per default/as an standard tool, the integration of the opposing jaw and occlusal record by means of virtual articulators for a continuous improvement in a complete digital workflow.

Using conventional protocols, abutments are planned after implant placement. Therefore, impressions are taken either conventionally or digitally to transfer implant positions to a model. In accordance with the planned prosthetic superstructure, the position of the inserted implants and the course and thickness of the existing periimplant gingiva, stock or individual abutments can be selected or manufactured individually.

Individual abutments are beneficial for esthetics because the shape of the emergence profile can be individually designed and adjusted with respect to the prosthetic set-up [28]. In case of an unfavorable abutment position, its visualization at the time of the prosthetic set-up and virtual implant planning helps to improve the implant position and selection of components. None of the tested software systems provided tools for the design of an individual abutment.

Stock abutments could be displayed in the prosthetic set-up after virtual implant planning in CoDiagnostiX, Simplant, Smop and ImplantStudio. Straight or angled abutments were modifiable regarding the prosthetic set-up and various sizes. To date, the selection of implants and corresponding abutments are very limited. Except for one software (CDX), none of the provided stock abutments was compatible with the used implant types in the present study. With NobelClinican, abutments could solely be displayed for Nobel Biocare implants. Although implant manufacturers such as Dentsply Sirona (Charlotte, NC, USA), Camlog Biotechnologies GmbH (Basel, Switzerland) and Institut Straumann AG (Basel, Switzerland) were available in the virtual implant planning software, a visualization of abutments was not possible.

This part of the narrative review focused on the prosthetic set-up and virtual implant planning in dental implant planning software. The accuracy of the transfer of implant positions using drill guides was not assessed, as it is dependent on factors such as clinical situation, drill guide support and drill protocols/instruments that may be selected independent of the software system. The time and cost related comparison was not drawn between systems, as they are based on the experience of the user with each system and might not be fully evaluated with the presented methodology. Two partially edentulous cases were selected to assess the possibilities and limitations of prosthetic set-up and implant planning in different software systems. The rationale for the selected cases was the inclusion of a single and multiple missing teeth, maxilla and mandible, interdental tooth gap and cantilever situation, respectively. The results related to partially edentulous cases do not apply for all clinical situations including fully edentulous jaws, as specific requirements may exist.

## Conclusions

All examined systems provided three-dimensional reconstructions or two-dimensional cross-sections with varying orientation for virtual implant placement. The databases differ between 7 and 120 implant systems available for the examined planning systems. The import of a virtual prosthetic set-up, the selection of different tooth designs or the use of a standard tooth shape was possible in all systems. Only one system (IST) allows the use of a virtual articulator. None of the tested implant planning systems provided tools for the design of an individual abutment. A higher compatibility with universal formats to import (e.g. face scans, scanner specific formats including color information) would increase the ease of use.

## Supplementary Information

**Additional file 1.** Current availability of implant manufacturers for the reviewed dental implant planning software.

## Data Availability

Not applicable.

## Abbreviations

CAD: Computer aided design
CAM: Computer aided manufacturing
CBCT: Cone beam computed tomography
CDX: CoDiagnostiX
DICOM: Digital imaging and communication in medicine
IST: Implant studio
NC: Nobel clinician
SIM: Simplant
SMP: Smop
STL: Standard tesselation language
