# Supplementary material for: Digital implantology—a review of virtual planning software for guided implant surgery. Part II: Prosthetic set-up and virtual implant planning
Source: BMC Oral Health. 2022 Jan 30;22:23. doi: 10.1186/s12903-022-02057-w (PMC8802526; doi:10.1186/s12903-022-02057-w)
Supplement: Supplementary file 1 — Additional file 1. Current availability of implant manufacturers for the reviewed dental implant planning software. [file 12903_2022_2057_MOESM1_ESM.docx]

Addendum: Availability of implant manufacturers for each planning software:

**CDX**

86 available implant manufacturers (Version 10.3)

| 3D Diagnostix Inc.  Alpha-Bio Tec  Anthogyr  Argon Medical  Aurosan GmbH  Avinent Implant System  BEGO Implant Systems  BioHorizons  Biologitech srl  Biomet 3i  Biotech Dental  Bredent Medical  CAMLOG  Cortex Dental  DenTack PYRAMIDION Dental Implant  Dental Tech S.r.l.  Dentalpoint AG  Dentegris GmbH  Denti System Ltd.  Dentium  Dentsply Friadent  Dérig  Ditron Dental  Easy System Implant  Euroteknika  FairImplant GmbH  FMZ GmbH  GC Tech  Geass srl  General Implants  Glidewell  Global D SAS  Hiossen  HI-TEC IMPLANTS LTD  Implant Direct  IMPLANTS DIFFUSION INTERTIONAL  Keystone Dental  Klockner  LASAK s.r.o.  LEONE Spa  Little Implant Co.  Medentika GmbH  medentis medical  Medical Instinct  Megagen Implant  MIS Implants Technologies Ltd.  Neodent  Neoss  Nobel Biocare  Nuvo™ | O.M.T.  OCO Biomedical Inc.  ORAL ICEBERG S.L.  Osstem Implant  OXY IMPLANT  Paltop Advanced dental Solutions  Plan 1 Health  Prodent Italia S.r.l.  Prowital GmbH  Rex Implants Inc.  Ritter Implants GmbH & Co. KG  S.I.N. IMPLANT SYSTEM  SGS Dental  Shinhung  SIC® invent AG  Southern Implants  SpiralTech  steco-system-technik  Sterngold Dental  Straumann  Sweden & Martina S.p.A.  Swiss Dental Solutions AG  T.A.G. Dental  TBR Implants Group  Test cylinder  TFI System Srl  Thommen Medical AG  Titaniumfix  TRATE AG  TRI Dental Implants Int. AG  Trinon Titanium GmbH  YDM  Zest Dental Solutions  Zimmer Dental  ZL Microdent  Z-Systems |
| --- | --- |

26 available for fully-guided (osteotomy alone OR osteotomy with implant placement)

| Alpha-Bio Tec GuidedSurgery  Anthogyr INITIAL / INTEGRAL  Argon Medical Rapid Surgery  BEGO Guide  BioHorizons  Biologitech  Biomet 3i Tapered Navigator®  Biotech Dental AtlaSurgery  bredent medical SKY pro guide  CAMLOG PL Guide System / Guide  Dentaurum  Hahn™ Tapered Implant Guide  Medentika MedentiGuide / MEDENTiKA Procone  MIS MGUIDE  Neodent / Neodent EasyGuide  Neoss  StecoGuide GuidedEndo  Straumann Guided Surgery  Sweden & Martina  TBR GUIDED CLASSIC / MCI | Thommen Medical Guided System / Pilot System  Titaniumfix  TRI Guided Surgery  YDM  Zimmer Guided Surgery |
| --- | --- |

**SIM**

120 available implant manufacturers (Version 18.5)

| Adin  Alpha Dent  Alpha-Bio  American Dental Implants  ANTHOGYR  AQB  Argon  ATOLL lmplant  Avinent  BB Dental  BEGO Implant Systems  BI  Bicon  Biocomp  BioHorizons  Biomed Implants  Biomet3i  BioSAFIN  Biotec Btk  Biotech  Bone System  BPI  BrainBase  Bredent medical  BTI  BTLock  C-Tech Implant  CeraRoot  CLC Scientific  Conmet  Dental Ratio  DENTALIS BIO SOLUTIONS  Dentatus  Dentaurum Implants GmbH  Dentegris  DENTIS  Dentium  DIO  Dr. Ihde  Dyna Implants  EasyGrip  Edierre Implant System  ESI  Euroteknika  FairImplant  FMZ GmbH  GC  Geass  Glidewell Laboratories  GLOBAL D  GMI  Hi-Tec  Hiossen  i-system  IAT  Implant Diffusion International  Implant Direct  IMTEC  Indi  Intra-lock | JDentalCare  KENTEC  Keystone Dental  Klockner Implant System  KLS Martin  KYOCERA Medical Corporation  LASAK  Leader  Leone  MDJ  Medentika  Medentis  Medical Instinct  Medical Production  Megagen  Microdent  MK GmbH  Multysystem  NDI Medical  Nemris  NEODENT  Novodent SA  OCO BIOMEDICAL  Omt  OsseoLink  Osstem  Osteo-Ti  Osteoplant  OT medical  Phibo  Platon  Prodent Italia  Proekcia  Prowital  Reuter systems  Rex Implants  RIEMSER AG  Ritter Implants  Salvin  SHOFU  SIC  Southern Implants  Sterngold  Sweden-Martina  Sybron Implant Solutions  Synthes  Tatum Surgical  TAV Dental  TBR  Ticare  TRI Dental Implants  Trinon  U-impl  Ustomed Instrumente  Victory  Warantec  Z-Systems  Zeramex  Zest Anchor  Zimmer Dental |
| --- | --- |

24 available for fully-guided (osteotomy alone OR osteotomy with implant placement)

| Astra Tech lmplant System TX / EV / EV Pilot  Xive  Ankylos  Biomet 3i Parallel walled / Tapered  Nobel Biocare  Straumann  Camlog  Anthogyr  SIC  Zimmer Dental  Ticare Fidelis  Global D  POI-EX  BTLock  BioTech AltaSurgery  Sweden&Martina  TFI EasyGrip / Smart  Geass Gealize / Geadrive  BEGO  Kentec | BioHorizons  Bicon  Klockner  MIS |
| --- | --- |

**SMP**

52 available implant manufacturers (Version 2.17)

| Alpha-Bio Tec  Anthogyr  B&B Dental  BIOMET 3i  BIOTECH INT.  BTI  BTK  Bego  Bicon  BioHorizons  Biodenta  Bredent Medical  C-TECH  CAMLOG  DENTSPLY  DIO  Demo  Dentaurum  Dentium  FairImplant  Futur Implant  GC Tech  GlobalD  JDental  Keystone Dental  Lasak  Leone  MIS  Medentika  Medentis  Medical Instinct  Megagen  Neodent  Neoss  Nobel Biocare  Osstem  Osteo-ti  Paris  SDS  SIC  Southern Implants  Steco  Straumann  Study Implants  Sweden & Martina  TAG Dental  TBR  TRATE AG  TRI  Thommen Medical | VITA  Zimmer |
| --- | --- |

36 available for fully-guided (osteotomy alone OR osteotomy with implant placement)

| Alpha-Bio Tec  B&B Dental  BIOMET 3i  BTK  Bego  Bicon  BioHorizons  Bredent Medical  C-TECH  CAMLOG  DENTSPLY  Dentaurum  Dentium  Futur Implant  GlobalD  JDental  Keystone Dental  Lasak  Leone  MIS | Medentika  Medentis  Megagen  Neodent  Neoss  Nobel Biocare  Osstem  SIC  Steco  Straumann  Sweden & Martina  TAG Dental  TBR  TRATE AG  Thommen Medical  Zimmer |
| --- | --- |

**NC**

7 available implant manufacturers (Version 3.5)

| Alpha-Bio Tec  CAMLOG  Dentsply  Implant direct  MIS  Nobel Biocare  Straumann |  |
| --- | --- |

1 available for fully-guided (osteotomy alone OR osteotomy with implant placement)

| Nobel Biocare |  |
| --- | --- |

**IST**

100 available implant manufacturers (Version 7.8.2.0)

| 3DCelo  3DDX  3Diemme  AB Dental  Adin  Alfa Gate  Alliance  Alpha Dent  Alpha-Bio Tec  Anthogyr  Argon  Avinent  B&B Dental  Bego  Bicon  Biodenta  BioHorizons  Bionmate  Biomet 3i  Biotech Dental  Biotem  BlueSkyBio  Bredent  BTI  BTLock  Camlog  Conexao  Cortex  Cowellmedi  CSM lmplant  C-Tech  DenTack  Dentatus  Dentaurum  Dentegris  Dentis  Dentium  Dentsply  ETGAR  Euroteknika  FGM  Geass  Global D  Hahn  Hiossen  lmplant Direct  lmplants Diffusion International  lnclusive Tapered  Intra-Lock  JDental Care | Just lmplant  Keystone  Kyocera  Lasak  Leader ltalia  Leone  Little lmplant Co.  Logon  Medentika  Megagen  MIS  Mozo Grau  Neobiotech  Neodent  Neoss  Nobel Biocare  NTA  OCO -Biomedical  Osstem  Paltop  Phibo  Point lmplant  Rex  Ritter Implants  RS Dental  SDS  SGS Dental  SIC  SIN  Singular lmplants  Southern lmplants  SpiralTech  Sterngold  Straumann  Sweden&Martnia  TAG  Taishan  TBR  Thommen  Titaniumfix  Trate  TRI  TruAbutment  URIS  Warantec  WEGO  Whitek  Zimmer  Z-systems  Zuga Medical |
| --- | --- |

66 available for fully-guided (osteotomy alone OR osteotomy with implant placement)

| 3DDX  3Diemme  Adin  Alfa Gate  Alliance  Alpha-Bio Tec  Argon  Avinent  B&B Dental  Bego  Bicon  BioHorizons  Bionmate  Biomet 3i  Biotech Dental  Bredent  Camlog  Conexao  Cortex  Cowellmedi  CSM lmplant  C-Tech  Dentaurum  Dentis  Dentium  Dentsply  Euroteknika  FGM  Geass  Hahn  Hiossen  lmplant Direct  lnclusive Tapered  Intra-Lock  JDental Care  Kyocera  Lasak  Leader ltalia  Leone  Medentika  Megagen  MIS  Mozo Grau  Neobiotech  Neodent  Neoss  OCO -Biomedical  Osstem  Phibo  RS Dental | SGS Dental  SIC  SIN  Singular lmplants  Straumann  Sweden&Martnia  TAG  TBR  Thommen  Titaniumfix  TRI  URIS  Warantec  Whitek  Zimmer  Zuga Medical |
| --- | --- |
